# Editorial: News and update in transoral robotic surgery in oropharyngeal, hypopharyngeal, and laryngeal cancers

**DOI:** 10.3389/fonc.2023.1199848

**Published:** 2023-05-18

**Authors:** Norhafiza Mat Lazim, Carmelo Saraniti, Barbara Verro

**Affiliations:** ^1^ Department of Otorhinolaryngology-Head and Neck Surgery, School of Medical Sciences, Universiti Sains Malaysia, Kubang Kerian, Kelantan, Malaysia; ^2^ Department of Biomedicine, Neurosciences and Advanced Diagnostics, University of Palermo, Palermo, Italy

**Keywords:** transoral robotic surgery, oropharyngeal carcinoma, laryngeal carcinoma, HPV positivity, airway obstruction

The Upper Aerodigestive Tract Tumour (UADT) is prevalent in our community, both in Western and Eastern world. Treatment of UADT tumour is challenging and hampered by multiple significant factors. This tumour has predilection for recurrence and have a high metastatic potential especially, stage III and IV tumours. Surgical resection of this tumour causes significant functional impairment as most of the time, the adjacent tissues and organs need to be resected together in order to achieve a negative surgical margin. In the narrow space area of the head and neck, the advent of minimally invasive surgery such as a robotic system escalates the efficiency of the therapeutic approach for this malignancy. To date, with newer refinement to the existing robotic systems, the efficiency of the surgical treatment and access of the UADT can be enhanced. Consequently, the outcome of optimal treatment can be achieved and less morbidities caused to the patients. This will improve patient’s overall quality of life.

Transoral Robotic Surgery (TORS) for UADT has been in practice for many years, as early as its inception in the early 2000s, where Dr Gregory Weinstein and Dr Bert O’Malley successfully used the Da Vinci robotic systems to resect the oropharyngeal tumours (1). Since then, there have been many scientific studies emerging that try to explore the effectiveness of this method in the head and neck oncology center worldwide. At present, this TORS technique has been shown to improve the outcome treatment of selected patients with the UADT tumours. This inclusive of recent studies on the application of TORS in the oropharyngeal and laryngeal surgery. Critically, there are many other multiple recent studies investigating the efficacy of TORS in preserving the functions of speech, swallowing and the quality of life of these patients with UADT tumours.

Generally, the status of HPV positivity of oropharyngeal carcinoma determines the final mode of treatment strategy. The HPV status also dictates the outcomes and prognosis of patients with oropharyngeal carcinoma. This however may differ within varied geographical locations, because of different tumour biology and patient characteristics. This necessitates other new detailed studies from different continents so that specific treatment outcomes can be measured and translated to local clinical practice. A bigger multi-institution collaborative study is highly desired at this stage, in order to have more accurate and better findings that can be highly relevant for future application.

This Research Topic encompasses use of TORS in 3 main UADT cancers. Specifically, each paper highlights the functional outcomes of TORS. In their systematic review, Lai et al. compared functional outcomes between TORS and endoscopic pharyngo-laryngeal surgery (EPLS) in patients affected by hypopharyngeal carcinoma. Both surgeries represent new organ-preserving surgical techniques that allow patients to achieve better quality of life post-surgical treatment. In particular, the study demonstrated that 136 out of 147 patients, who underwent TORS, didn’t have swallowing disorders (dysphagia and/or aspiration). Post-operative bleeding occurred only in 8 cases. Importantly, the rate of post-operative complications was similar between TORS and EPLS, without statistical difference for bleeding and swallowing disorders.

Additionally, Sano et al. analysed functional outcomes between TORS, EPLS and transoral videolaryngoscopic surgery (TOVS) showing their advantages as minimally invasive surgeries for pharyngeal and laryngeal tumours. All of the three therapeutic techniques have a low rate of post-operative complications, such as bleeding and emergency tracheostomy. In particular, several studies included in this review stressed that TORS is related to a lower risk of permanent gastrostomy and/or tracheostomy as well as lower rate of positive resection margins than other surgical therapies. These advantages lead to a better quality of life for patients that is one of the main goals of minimally invasive surgery, such as TORS.

Another study pointed out the post-operative mortality risk after TORS due to pT1-T2 oropharyngeal squamous cell carcinoma. This retrospective study by Davies et al. revealed that the risk of 30-day post-op mortality was very low (about 0.6%) and that it was higher in elderly patients (over 65 years old). These results suggest the effectiveness of TORS among the therapeutic protocols in early oropharyngeal cancer taking into account the strict patient selection in terms of oropharyngeal exposure and age. Indeed, the authors argued that these determinants could increase the risk of peri-operative complications and the rate of positive resection margins.

Wang et al. studied and analysed the role of TORS for early glottic cancer involving anterior commissure (AC). Indeed, AC represents a tricky laryngeal subsite due to its close continuity with thyroid cartilage, promoting its early infiltration. Eckel et al. demonstrated that cancer involving AC had higher risk of local recurrence than other laryngeal sites (2). In their study, Wang et al. reported a good oncological and functional outcome after TORS for AC cancers with 5-year overall survival of 93.8% and recurrence-free survival of 74.6%, without post-operative complications, and with satisfactory swallowing and voice outcomes. So, TORS represents a highly valuable surgical option for resection of early glottic cancer.

Critically, all the studies included in this topic have stressed the main limit of TORS, which includes the need of an optimal visualization and exposure of the operating field. Indeed, reduced mouth opening, trismus, retrognathia and/or big tongue represent relative contraindications to TORS.

Generally speaking, the treatment of HPV positive oropharyngeal carcinoma with TORS is advocated for early-stage tumours, i.e. stage I and II tumours. This allows tumour resection with negative margins, provided the wide oropharyngeal exposure can be obtained. Selection of patients is vital to ensure success using TORS, for instance patients with trismus is a contraindication for these techniques. Despite the similar survival outcomes for chemoradiation and TORS for early-stage tumour, TORS is preferable as it can negate the morbidities of chemoradiation which most of the time is significant and debilitative.

The elegant TORS have been compared with many other minimally invasive surgeries. The reported benefit of TORS includes reduced positive surgical margins of oropharyngeal cancer resection, which is vital in head and neck oncology. This however needs to be closely examined, as the patient’s selection criteria is limited to early-stage T1-T2 tumour. Many more studies are desired in order to confirm this finding in the near future.

The cost of TORS in very high and is cumbersome to institution from developed countries. Thus, not many centres are able to utilize TORS in head and neck cancer management. Indeed, the learning curves of TORS is long headed, and many more aspects need improvement. Persistent practices and training of young head and neck surgeons is imperative in coming years, as well as the finesse of TORS that can be implemented in order to achieve the best treatment outcome for the patients.

## Author contributions

All authors listed have made a substantial, direct, and intellectual contribution to the work and approved it for publication.
